# Effect of Psyllium on Physical Properties, Composition and Acceptability of Whole Grain Breads

**DOI:** 10.3390/foods11121685

**Published:** 2022-06-08

**Authors:** Maria Franco, Manuel Gómez

**Affiliations:** College of Agricultural Engineering, University of Valladolid, 34004 Palencia, Spain; mariafrancomarcos@gmail.com

**Keywords:** psyllium, wholemeal bread, mixolab, acceptability, nutrition

## Abstract

Despite the clear nutritional advantages of wholemeal breads, their consumption is lower than recommended, mainly due to their lower organoleptic quality. This paper proposes the use of psyllium to improve the quality of these breads. For this aim, a wholemeal bread control is compared to breads with psyllium added in different amounts (1 to 10%). Mixolab was used to analyse dough behaviour. Specific volume, texture, macronutrient composition, and bread acceptability were also analysed. Increasing amounts of psyllium resulted in an increased dough hydration and stability, but a reduced kneading time. Specific volume and weight loss were not affected, despite the higher hydration level of the doughs. The addition of psyllium reduced bread hardness and increased its cohesiveness and resilience, thus lowering staling. The addition of psyllium also reduced the calorie content of the breads, due to increased moisture and fibre content. Moreover, the addition of up to 5% psyllium clearly improves the acceptability of wholemeal breads. The use of psyllium can improve the organoleptic and nutritional quality of wholemeal breads, improving their acceptability by consumers.

## 1. Introduction

Bread is a staple food for humans as well as the main source of carbohydrates in the population of many countries. Increased consumption of whole grains is recommended as part of a healthy and sustainable diet because it is associated with a lower incidence of and mortality from CVD, type 2 diabetes, and some cancers [1]. The higher fibre content of wholemeal breads compared to white wheat breads is evident in their composition [2]. A minimum intake of 125 g/day of whole grains and 24 g/day of fibre is recommended. In fact, increased consumption of whole grains and fibre are two of the main dietary actions that could most effectively reduce people’s mortality [3,4]. However, consumption of whole grains is low, mainly due to their lower sensory quality and a lack of knowledge on how to process them [5,6].

Xanthan gum has been proposed as an improver in the production of white bread [7]. This hydrocolloid has also been proposed to improve the quality of wholemeal breads because it increases water absorption and specific volume and reduces their hardness [8]. Psyllium is a natural product with a high-water absorption capacity and has a similar effect to xanthan gum when combined with starchy substances [9]. Psyllium gum is a hydrocolloid found in the husk of seeds from *Plantago ovata*. The main global manufacturer of these seeds is India, but production in Pakistan and Iran is also important. Psyllium husk is mainly composed of fibre, in particular arabinoxylans. Aspects of the composition, functionality and possible applications of psyllium can be consulted in the review of Belorio and Gómez [10]. The beneficial health effects of psyllium consumption have been widely studied and include the prevention of constipation, diarrhoea, irritable bowel syndrome (IBS), inflammatory bowel disease-ulcerative colitis, colon cancer, diabetes and hypercholesterolemia. These topics have been addressed in excellent literature reviews [11,12].

Psyllium has been used in the production of gluten-free breads as a gluten replacer [13,14,15,16]. However, studies on the use of psyllium in white bread are scarce [17,18,19], and there is no study on the use of psyllium as an improver in wholemeal bread.

The aim of this study is to analyse the effect of the addition of psyllium, in percentages of 1, 2, 5 and 10%, to wholemeal bread doughs. The effect on the rheology of the dough has been analysed using the Mixolab. Bread specific volume, weight loss, texture, macronutrient composition and sensory acceptability have been analysed.

## 2. Materials and Methods

### 2.1. Materials

Breads were elaborated using wholemeal flour (WF) supplied by Molinos del Duero—Carbajo Hermanos S.A. (Zamora, Spain). Wholemeal flour contains all parts of the grain, bran, germ, and endosperm, as present in the native state. Wholemeal flour content was 14.6 g/100 g water, 13.6 g/100 g protein, 9.3 g/100 g fibre and 1.5 g/100 g ash (data provided by the manufacturer). Psyllium (PSY) (VITACEL^®^ Psyllium P95) was provided by J. Rettenmaier & Söhne GmbH & Co. KG (Rosenberg, Germany) and was used as an improver. This commercial product is obtained by milling psyllium husk. Other ingredients were instant yeast (Dosu Maya Mayacilik A.S., Istanbul, Turkey), refined salt (Disal, Madrid, Spain), and potable water.

### 2.2. Methods

#### 2.2.1. Dough Rheology

Rheological properties of doughs were performed using the Mixolab (Chopin, Tripette et Renaud, Villeneuve La Garenne, France) and the standard AACC method 54-60.01 [20]. The Mixolab was used to measure the rheological properties of doughs subject to the dual stress of mixing and temperature changes.

Analysis was performed in duplicate.

#### 2.2.2. Bread Making

Breads were elaborated using a straight method and all the ingredients were calculated on a g/100 g flour basis. A control bread (Control) was elaborated, using WF. Psyllium (1, 2, 5, and 10 g/100 g) was added by flour replacement in each formulation to produce: PSY 1%, PSY 2%, PSY 5%, and PSY 10%. The water content of the formulations was 95% of the mixolab water absorption values; 75% (WF), 78,9% (PSY 1%), 85% (PSY 2%), 100% (PSY 5%), and 125% (PSY 10%). Water temperature was calculated to achieve a final dough temperature of 23 °C. The ingredients, including flour, psyllium, instant dry yeast (2 g/100 g) and salt (1.8 g/100 g), were placed together, and then, water was added. All ingredients were kneaded in a spiral mixer (Ferneto, Vagos, Portugal) until the gluten network was fully developed. Pieces of 300 g were obtained and manually rounded. After allowing these to rest for 10 min, bars (230 mm long) were formed using a bar former (Subal, Paterna, Spain), some parts were placed in aluminium moulds (232 mm × 60 mm × 108 mm), and some other parts were formed into bars outside the mould for fermentation. The fermentation was conducted for 120 min at 30 °C and 75% of relative humidity. Breads were baked in a convection oven (Rotoram, Ramalhos, Agueda, Portugal) at 220 °C for 25 min.

After baking, the breads were left to cool for 1 h at room temperature. Finally, the samples were packed in plastic bags, duly sealed, and stored for 24 h until later analysis. All bread formulations were performed in duplicate (two batches).

#### 2.2.3. Bread Characteristics

The height and width of the breads were measured at the centre of the sample using a digital calliper (bars outside the mould). The weight loss was measured as the difference between the weight of the dough before baking and the weight of the breads 24 h after baking, and the results were expressed as a percentage (bars in moulds). The final volume of the bread (bars in moulds) was obtained with a Volscan Profiler volume analyser (Stable Microsystems, Surrey, UK). The specific volume of the bread was calculated as the ratio of bread volume to bread weight (cm^3^/g). All measurements were performed on four samples of each elaboration.

Crumb texture (bars in moulds) was measured through a texture profile analysis (TPA) with a double compression test using a TA-XT2 texture analyser (Stable Microsystems, Surrey, UK). A 30 mm thick middle loaf was evaluated from four breads of each preparation (1 × 4 × 2). For the analysis, a 25 mm diameter cylindrical probe penetrated 50% of the depth of each slice using a trigger force of 5 g and a test speed of 1 mm/s crumb. The application conditions of the TPA method were as follows: pre-test speed 2 mm s^−1^, test speed 1 mm s^−1^, and post-test speed 2 mm s^−1^. Hardness, cohesiveness, chewiness and resilience were obtained [21]. Crumb hardness was also evaluated seven days after baking to obtain the increase of hardness (Δ), which was calculated as the difference between the results of hardness at day seven and day one.

#### 2.2.4. Proximate Composition

For macronutrients analysis, determination of moisture content was determined by moisture oven rying [22]. Total fat was analysed by Fat Soxhlet analyser (Soxtec 8000, FOSS, Hilleroed, Denmark) according to Shin et al. [23]. Total protein was determined by the Kjeldahl method [24], ash by the incineration method [25], and dietary fibre by the enzymatic method [26]. Other carbohydrates have been calculated by difference.

#### 2.2.5. Sensory Analysis

Consumer test was performed by 108 volunteers (71 females and 37 males), regular bread eaters, aged 18–65 years recruited at the university campus (Palencia). All respondents have consented to participation in the study. Breads were evaluated based on consumer acceptance of their appearance, odour, texture, taste, and overall acceptability, using a hedonic scale ranging from “I dislike very much” (1 point) to “I like very much” (9 points). The consumers carried out the test in individual booths, with natural light, absence of external noise, and with a temperature between 20–24 °C. Samples of control, PSY 2%, PSY 5%, and PSY 10% were divided into 5 cm-wide slices and presented on white paper plates coded with four-digit numbers and served in random order. Consumers were also able to look at a whole loaf of bread to assess the acceptability of the appearance. Water was available for rinsing. The sensory analysis was approved by the research ethics of the health area of Palencia (registration n° 2019/026).

#### 2.2.6. Statistical Analysis

Analysis of variance (ANOVA) was used to study the differences between dough and bread characteristics. Tukey’s HSD was used to describe means with 95% confidence. Analysis was performed using Statgraphics Centurion 18 V18.1.13 software (StatPoint Technologies, Warrenton, VA, USA).

## 3. Results and Discussion

### 3.1. Dough Rheology

**Figure 1:**
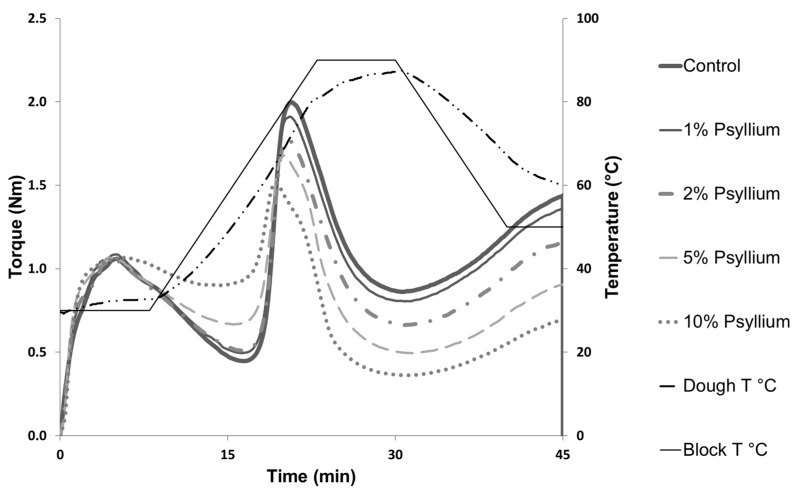
Mixolab curves. Control: whole wheat flour; PSY 1%: whole wheat flour with 1% psyllium; PSY 2%: whole wheat flour with 2% psyllium; PSY 5%: whole wheat flour with 5% psyllium; PSY 10%: whole wheat flour with 10% psyllium.

Dough absorption increases as the amount of psyllium increases (Table 1); it increases by 67% when 10% of psyllium is incorporated, result that is in accordance with psyllium’s high water absorption capacity [10]. In fact, it agrees with observations made by other researchers who conducted farinographic analysis incorporating hydrocolloids, such as xanthan gum, with similar functionality to psyllium [7,9], psyllium itself, in doughs made with mixtures of corn starch and gluten [27], or wheat flours [28]. The need to increase the hydration of the doughs when the psyllium content is increased has also been observed in gluten-free breads [29]. Figure 1 shows the behaviour of the doughs during kneading (first part of the curve) and during baking (second part). As it can be observed, in the first part of the analysis, psyllium incorporation reduces the time needed to develop the gluten network and gives greater stability to the doughs. These changes are most visible at 5% and increase as the psyllium dose does. The shorter kneading time may be due to the lower flour content of the mixes and, therefore, the lower gluten content. The higher stability could be explained by the fact that the consistency of the dough is partly achieved by the thickening power of psyllium. As a consequence, the usual weakening of wheat doughs due to a degradation of the gluten network is minimised. Rosell, Rojas and Barber [7] also observed, using a farinographic analysis, a greater stability of the doughs with the addition of xanthan gum, but this increased the development time. The differences can be explained by the higher hydration of the doughs in the mixolab. In fact, with the mixolab, Rosell, Collar, and Haros [30] did not observe any effect of xanthan gum on these parameters. It should be noted that in these studies the maximum dose of xanthan added was 0.5%, which is lower than the psyllium dose used in our study.

On the second part of the curve, it can be seen that the psyllium doughs have a lower peak, and a lower torque during heating and subsequent cooling. This may result in a softer texture of the breads produced. Starch is the flour component responsible for the increase in dough viscosity during heating when it gelatinises [31]. Therefore, it could be expected that when psyllium is incorporated, the flour content, and consequently the starch content, is reduced, resulting in a reduction in the consistency of the dough at this stage. However, in previous works with the Rapid Visco Analyser (RVA) and gluten-free starches or flours, either an increase in viscosity or little effect is observed with the addition of psyllium after gelatinisation [9,32,33]. This suggests that the thickening power of psyllium under these conditions is similar to or even higher than that of gelatinised wheat starch. It is therefore more accurate to think of the possible competition of psyllium and starch for water, due to the high water absorption capacity of psyllium, as stated by BeMiller [34] about other hydrocolloids. That would explain why in the RVA analysis this effect is not noticeable, as a higher amount of water was used and, therefore, water was available. However, in the mixolab, where a much lower water content is used, this effect is clearer. These differences in pasting properties as a function of starch/water ratio have already been observed by Doublier, Llamas, and Lemeur, [35]. It should be noted that mixolab hydration is more similar to baking hydration and, therefore, more useful for predicting the properties of the bread obtained.

### 3.2. Bread Quality

**Figure 2:**
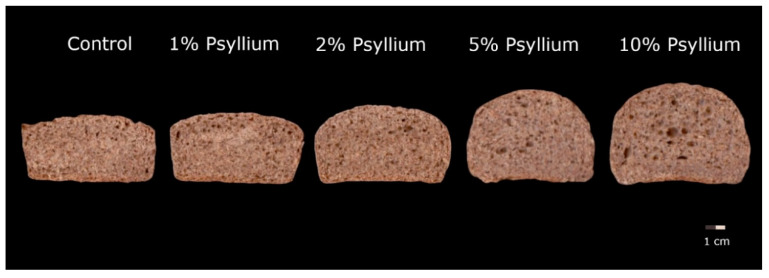
Image of the central slices of control and psyllium added whole breads. Control: whole wheat flour; psyllium 1%: whole wheat flour with 1% psyllium; psyllium 2%: whole wheat flour with 2% psyllium; psyllium 5%: whole wheat flour with 5% psyllium; psyllium 10%: whole wheat flour with 10% psyllium.

No significant difference has been observed in the specific volume of the breads, or in weight loss during baking, with the incorporation of psyllium. Therefore, in spite of adding more water during the kneading process, the volume of the breads is not reduced, and the excess water is not lost in the baking process, so the final breads will be moister and juicier. In Figure 2, the central slices of the psyllium breads are higher, but a drop in height can also be observed as we approach the ends of the breads. In this case, moisture adjustment is very important, because if the water content is not increased when psyllium is incorporated, there is a significant reduction in the specific volume [17]. However, differences in the height/width ratio are observed when breads are fermented and baked outside of a mould. This ratio increases as psyllium is incorporated. This indicates that the incorporation of psyllium in these percentages helps maintain the shape given to the dough after kneading and to achieve rounder, less falling and less flattened breads. Farbo et al. [16] observed that the incorporation of 1% psyllium did not break the gluten network, unlike other hydrocolloids, and helped reinforce it by increasing fermentation tolerance and gas retention in fermentation. Combined with the higher tolerance observed in the mixolab analysis, this would explain the higher height/width ratios of the psyllium breads.

In terms of bread texture (Table 2), the incorporation of psyllium reduces bread hardness and chewiness and increases cohesiveness and resilience. The increase in hardness observed during bread storage is also reduced, improving shelf life. This improvement in shelf life was already observed by Fratelli et al. [36] when studying gluten-free breads enriched with psyllium. In most of these parameters, to detect significant differences compared to the control, it is necessary to reach levels of 5%. The fall in hardness may be related to the lower consistency of the dough after heating observed in the mixolab, because this process simulates baking. This may be due partly to the lower starch content, which is responsible for crumb hardness after starch gelatinisation and retrogradation phenomena, and partly for the higher moisture content of the breads, as no significant differences in weight loss were observed. Biliaderis, Izydorczyk, and Rattan [37], also observed a reduction in bread hardness when the content of arabinoxylans, which is the main component of psyllium, was increased. Water was thought to be responsible for this effect as it acts as a plasticiser in the gluten–starch composite matrix decreasing the stiffness in the final product. The reduction in hardness may also be due to a lower starch content, as this hardness is partly due to amylopectin retrogradation phenomena, together with changes in moisture redistribution [38]. Therefore, it may also indicate better water retention and, therefore, less drying out of the breads. In fact, Filipcev, Pojic, Simurina, Misan, and Mandic [13] attributed the ability of psyllium to reduce the staling of gluten-free breads to the reduction in easily removable water and the binding strength of stronger bound water populations, observed by thermogravimetric analysis. Tebben and Li [8] observed similar results with the addition of xanthan gum in wholemeal breads. Although xanthan gum decreased amylose–lipid complexation, these researchers also attributed those results to the increased water retention capacity, as it did not affect amylopectin retrogradation.

### 3.3. Proximate Composition

Breads have higher values for moisture and fibre, and lower values for protein, ash and non-fibre carbohydrates with increasing psyllium content (Table 3). In most cases, significant differences could only be observed at 5% and above. As a result, breads with psyllium have a lower calorie content, achieving a reduction of 20.4% in the case of breads with 10% psyllium. Lower protein, ash and carbohydrate contents are due to the dilution of all components by the higher moisture content of the breads. The higher moisture content results from both the higher percentage of water in psyllium formulations and the high water absorption capacity of psyllium, which minimises water loss during baking and storage. This was proven by the absence of differences in water loss during baking, indicating that the excess water in the formulation, compared to the control, was retained in the bread. The higher fibre content results from psyllium being mostly composed of arabinoxylan-type fibres [39]. This agrees with a previous work [18], although it is not as high as expected due to the dilution effect of the higher moisture content of the final bread. Therefore, the breads obtained are not only good as wholemeal products [1], but they would also have a lower calorie content, a higher fibre content [4], and, in addition, would offer the health benefits associated with an increased psyllium intake [11,25].

### 3.4. Acceptability

Usually, a major problem with healthy breads is that their acceptability is often low. Table 4 shows that the incorporation of psyllium improved the taste and texture of wholemeal breads, increasing their overall rating when incorporated at 2 or 5%. In the case of breads with 5%, a value of more than 7 points, on a scale of 1 to 9, was achieved. This higher acceptability may be due to the dilution of bitter components, one of the reasons for the lower acceptability of wholemeal breads [40]. However, their higher acceptability may also be related to the reduction in the denser crumb texture typical of wholemeal breads [6] due to their higher water content. This higher water content gives them a greater juiciness which is appreciated by consumers in their comments. The major differences between the assessment of the control and the psyllium breads are in taste and texture. However, breads with 10% psyllium have a lower appearance rating, due to some crust wrinkling, which makes their overall rating lower than those with 5%, and no significant differences are observed with the control or PSY 2%. It should be noted that the overall rating of the 10% breads shows a higher variability than the other psyllium breads. This is because, while some tasters indicated that they were the highest-rated breads, because of their juiciness and softer texture, others rated them negatively for their appearance and for being too soft. This suggests that this type of bread may have a more specific target, in consumers who value juiciness or the absence of bitter flavours more than the external appearance of the breads. It should also be noted that the tasters were not given any nutritional information about the breads. Providing adequate nutritional information, highlighting the lower calorie values of psyllium breads, their higher fibre content, and the advantages of consuming psyllium and wholegrain products, could further increase the value of psyllium breads, as has already been demonstrated with beta-glucan breads [41].

## 4. Conclusions

With the addition of psyllium, it is possible to obtain breads with a higher volume and a specific volume similar to wholemeal breads, as well as more rounded shapes when baked out of a pan, significantly increasing the hydration of the dough. As the amount of psyllium is increased, the hardness is reduced, the cohesiveness of the crumb is increased, the fibre content is increased, the caloric content is reduced, and the acceptability of the breads is increased. The increase in overall acceptability is only observed up to 5%, as higher percentages may have a negative effect on overall acceptability due to the low evaluation of the appearance of the breads. The incorporation of psyllium can improve the acceptability and shelf-life of wholemeal breads and facilitate an increase in their consumption, improving their already highly interesting nutritional characteristics. However, the higher water content of breads with psyllium could facilitate the development of moulds, an aspect that must be taken into account if this type of bread is to be marketed

## Figures and Tables

**Table 1:** Control and psyllium added whole bread dimensions.

Flour	Weight Loss (%)	Specific Volume (cm^3^/g)	Bread Height/Width (mm)
Control	0.18 ± 0.00 a	2.14 ± 0.07 a	0.23 ± 0.01 a
PSY 1%	0.18 ± 0.02 a	1.97 ± 0.16 a	0.35 ± 0.09 ab
PSY 2%	0.17 ± 0.01 a	1.98 ± 0.11 a	0.42 ± 0.07 bc
PSY 5%	0.17 ± 0.01 a	2.16 ± 0.08 a	0.59 ± 0.05 bc
PSY 10%	0.18 ± 0.0 a	2.20 ± 0.06 a	0.68 ± 0.09 c

Control: whole wheat flour; PSY 1% whole wheat flour with 1% psyllium; PSY %: whole wheat flour with 2% psyllium; PSY 5%: whole wheat flour with 5% psyllium; PSY 10% whole wheat flour with 10% psyllium. Values with the same letter in the same parameter do not present significant differences (*p* < 0.05).

**Table 2:** Control and psyllium added whole bread texture properties.

Flour	Hardness (N)	Cohesiveness	Resilience	Chewiness (N)	Δ Hardness (%)
Control	16.15 ± 2.31 b	0.79 ± 0.02 ab	0.47 ± 0.01 a	11.73 ± 1.50 c	12.75 ± 1.50 b
PSY 1%	16.42 ± 3.48 b	0.78 ± 0.01 a	0.48 ± 0.01a	11.84 ± 2.61 c	12.79 ± 2.50 b
PSY 2%	12.76 ± 1.50 b	0.80 ± 0.00 ab	0.51 ± 0 ab	9.66 ± 1.23 bc	10.16 ± 1.20 b
PSY 5%	5.88 ± 0.39 a	0.82 ± 0.00 bc	0.53 ± 0.01 bc	5.77 ± 1.84 ab	4.84 ± 0.33 a
PSY 10%	2.98 ± 0.43 a	0.84 ± 0.025 c	0.55 ± 0.02 c	3.98 ± 0.08 a	2.50 ± 0.29 a

Control: whole wheat flour; PSY 1% whole wheat flour with 1% psyllium; PSY %: whole wheat flour with 2% psyllium; PSY 5%: whole wheat flour with 5% psyllium; PSY 10% whole wheat flour with 10% psyllium. Values with the same letter in the same parameter do not present significant differences (*p* < 0.05).

**Table 3:** Proximate composition of control and psyllium added whole breads.

Flour	Moisture	Ashes	Protein	Fats	Fibre	Other Carbohydrates
Control	39.53 ± 2.41 a	2.28 ± 0.05 b	8.62 ± 0.02 e	<0.1	7.83 ± 0.10 a	41.74 ± 2.74 c
PSY 1%	38.55 ± 0.53 a	2.28 ± 0.04 b	8.38 ± 0.08 d	<0.1	8.62 ± 0.22 ab	42.17 ± 0.63 c
PSY 2%	40.28 ± 0.40 a	2.16 ± 0.03 b	8.10 ± 0.00 c	<0.1	9.31 ± 0.08 bc	40.15 ± 0.19 c
PSY 5%	44.77 ± 0.75 b	1.80 ± 0.09 a	7.16 ± 0.01 b	<0.1	9.33 ± 0.92 bc	36.94 ± 0.26 b
PSY 10%	50.10 ± 0.62 c	1.72 ± 0.01 a	6.30 ± 0.01 a	<0.1	9.93 ± 0.39 c	31.95 ± 1.03 a

Control: whole wheat flour; PSY 1% whole wheat flour with 1% psyllium; PSY %: whole wheat flour with 2% psyllium; PSY 5%: whole wheat flour with 5% psyllium; PSY 10% whole wheat flour with 10% psyllium. Values with the same letter in the same parameter do not present significant differences (*p* < 0.05).

**Table 4:** Sensory acceptability of control and psyllium added breads.

Flour	Appearance	Odour	Taste	Texture	Overall Acceptability
Control	7.27 ± 1.37 b	6.76 ± 1.37 a	6.19 ± 1.55 a	5.74 ± 1.76 a	6.45 ± 1.38 a
PSY 2%	7.26 ± 1.13 b	6.85 ± 1.35 a	6.75 ± 1.37 b	6.74 ± 1.42 bc	6.87 ± 1.08 b
PSY 5%	7.18 ± 1.22 b	6.85 ± 1.35 a	6.93 ± 1.37 b	7.10 ± 1.48 c	7.24 ± 1.09 c
PSY 10%	5.97 ± 1.88 a	6.93 ± 1.17 a	6.65 ± 1.59 b	6.55 ± 1.85 b	6.76 ± 1.47 ab

Control: whole wheat flour; PSY 2% whole wheat flour with 2% psyllium; PSY 5%: whole wheat flour with 5% psyllium; PSY 10% whole wheat flour with 10% psyllium. Values with the same letter in the same parameter do not present significant differences (*p* < 0.05).

## Data Availability

The data presented in this study are available on request from the corresponding author.
